# Role of floral organ identity genes in the development of unisexual flowers of *Quercus suber* L.

**DOI:** 10.1038/s41598-017-10732-0

**Published:** 2017-09-04

**Authors:** Rómulo Sobral, M. Manuela R. Costa

**Affiliations:** Biosystems and Integrative Sciences Institute (BioISI), Plant Functional Biology Center, University of Minho, Campus de Gualtar, 4710-057 Braga, Portugal

## Abstract

Monoecious species provide an excellent system to study the specific determinants that underlie male and female flower development. *Quercus suber* is a monoecious species with unisexual flowers at inception. Despite the overall importance of this and other tree species with a similar reproductive habit, little is known regarding the mechanisms involved in the development of their male and female flowers. Here, we have characterised members of the ABCDE MADS-box gene family of *Q. suber*. The temporal expression of these genes was found to be sex-biased. The B-class genes, in particular, are predominantly, or exclusively (in the case of *QsPISTILLATA*), expressed in the male flowers. Functional analysis in Arabidopsis suggests that the B-class genes have their function conserved. The identification of sex-biased gene expression plus the identification of unusual protein-protein interactions suggest that the floral organ identity of *Q. suber* may be under control of specific changes in the dynamics of the ABCDE model. This study constitutes a major step towards the characterisation of the mechanisms involved in reproductive organ identity in a monoecious tree with a potential contribution towards the knowledge of conserved developmental mechanisms in other species with a similar sex habit.

## Introduction

Development of separate male and female flowers in the same individual (monoecy) or in different individuals (dioecy) is a highly adaptive trait that enhances cross-pollination and gene fluidity^1^. Flowers can become unisexual after floral organ specification due to carpel or stamen abortion or arrest. Other unisexual flowers are derived from floral meristems that fail to initiate female or male organ primordia, being unisexual at inception^2–7^. In these flowers, the likely sex-determinant genes should control the mechanisms between floral meristem initiation and floral organ identity^8^.

Development of floral organs has been extensively studied in hermaphrodite species where floral organ identity is controlled by well-described gene hubs, which in the majority of the cases, code for MADS-box transcription factors^9^. Detailed analysis of the genetic mechanisms controlling flower organ identity led to the proposal of the ABCDE model, in which different classes of genes are recruited in the flower meristem to specify the identity of non-reproductive (sepals and petals) and reproductive organs (stamens, carpels)^10–15^. In *Arabidopsis thaliana* and other hermaphrodite species, A- and E-class genes control sepal identity and A- combined with B- and E-class genes control petal identity. B- combined with the C- and E-class genes specify stamen identity, and C- and E- and D-class genes specify carpel identity. The ABCDE model has been successfully used to explain flower organ organisation in core eudicots in what seems to be a conserved mechanism^16–19^.

The *sliding boundaries* model^20, 21^ and the *fading borders* model^22^ are modified ABCDE models for the development of flowers of lower eudicots, monocots and basal angiosperms. Both models suggest that the unconventional flower phenotypes of these species are mainly due to alterations in the boundaries of the expression domains of B- and C-class genes. The differential regulation of B and C-class genes has been associated to the development of unisexual flowers by inception in some dioecious species^6, 7, 23^. In the dioecious *Spinacea oleracea*, B-class genes are unique or differentially expressed in the male flowers. In transgenic *S. oleracea* plants, down-regulation of a B-class gene in male flowers originates a conversion of male into female flowers^7^. Similar results were observed in *Thalictrum dioicum*, where targeted silencing of a B-class gene by virus-induced gene silencing resulted in homeotic conversion of the male flower into a female flower^23^. These results suggest that the expansion of B-class gene expression to the carpel whorl in male flowers might be preventing the development of the carpel. Still, there is not enough experimental evidence to explain how potential sex-determinant genes control the regulation of B or C- class expression in the early stages of floral organ determination of unisexual flowers by inception.

Flower unisexuality by inception is particularly common in monoecious tree species such as *Carya illinoiensis* (pecan), *Castanea sativa* (chestnut), *Juglans regia* (walnut), *Corylus avellana* (hazelnut), Quercus *spp*., Betula *spp*., *Platanus occidentalis, Persea Americana* (avocado) or *Cocos nucifera* (coconut)^24^. Despite the economic and ecological importance of these species little is know on the genetic mechanisms controlling their male and female unisexual flower development. *Quercus suber* L. is a perennial monoecious evergreen oak species abundant in the Mediterranean basin in savannah-type ecosystems. *Quercus suber* male flowers are contained in catkins that emerge on the branches of the previous growth season. Each individual catkin contains 15 to 25 staminate flowers without any evidence of aborted gynoecia^2^. Female inflorescences arise in spikes on the axils of new leaves containing three to eight individual flowers, that also do not show any morphological evidence of aborted male organs^2^, suggesting that both *Q. suber* male and female flowers are unisexual at inception.

Functional studies in a non-model tree species such as *Q. suber* are difficult to perform due to several limitations common to many non-model species: unsequenced genome, not amenable to genetic manipulation and a long life cycle. Recently, an high-throughput transcriptomic study was published compiling normalised transcriptomic profiles of several *Q. suber* organs, including the male and female flowers^25^. Another RNAseq study revealed the transcriptomic differences between the male and female floral programs at different developmental stages^26^ and provided a valuable tool for further molecular characterisation of the mechanism controlling flower development of this monoecious species. By comparing female and male non-normalized libraries, Rocheta and colleagues (2014) identified several homologues of transcription factors that showed differential expression. Amongst these were MADS-box transcription factors, thus suggesting a putative role in male and female flower organ identity in *Q. suber*.

The main objective of this study was to study the potential involvement of MADS-box genes in floral organ identify of the *Q. suber* flowers, as unisexual flower development may be correlated to readjustments in gene expression programs in the different floral primordia. *Q. suber* MADS-box homologues genes were identified from available *Q. suber* cDNA libraries by phylogenetic profiling and its expression determined in male and female flowers at different developmental stages. Protein-protein interaction analysis allowed the identification of putative protein-protein complexes that may be controlling different aspects of flower development including meristem determinacy and reproductive organ identity.

## Results

### The MADS-box family of genes is conserved in *Q. suber*

**Figure 1:**
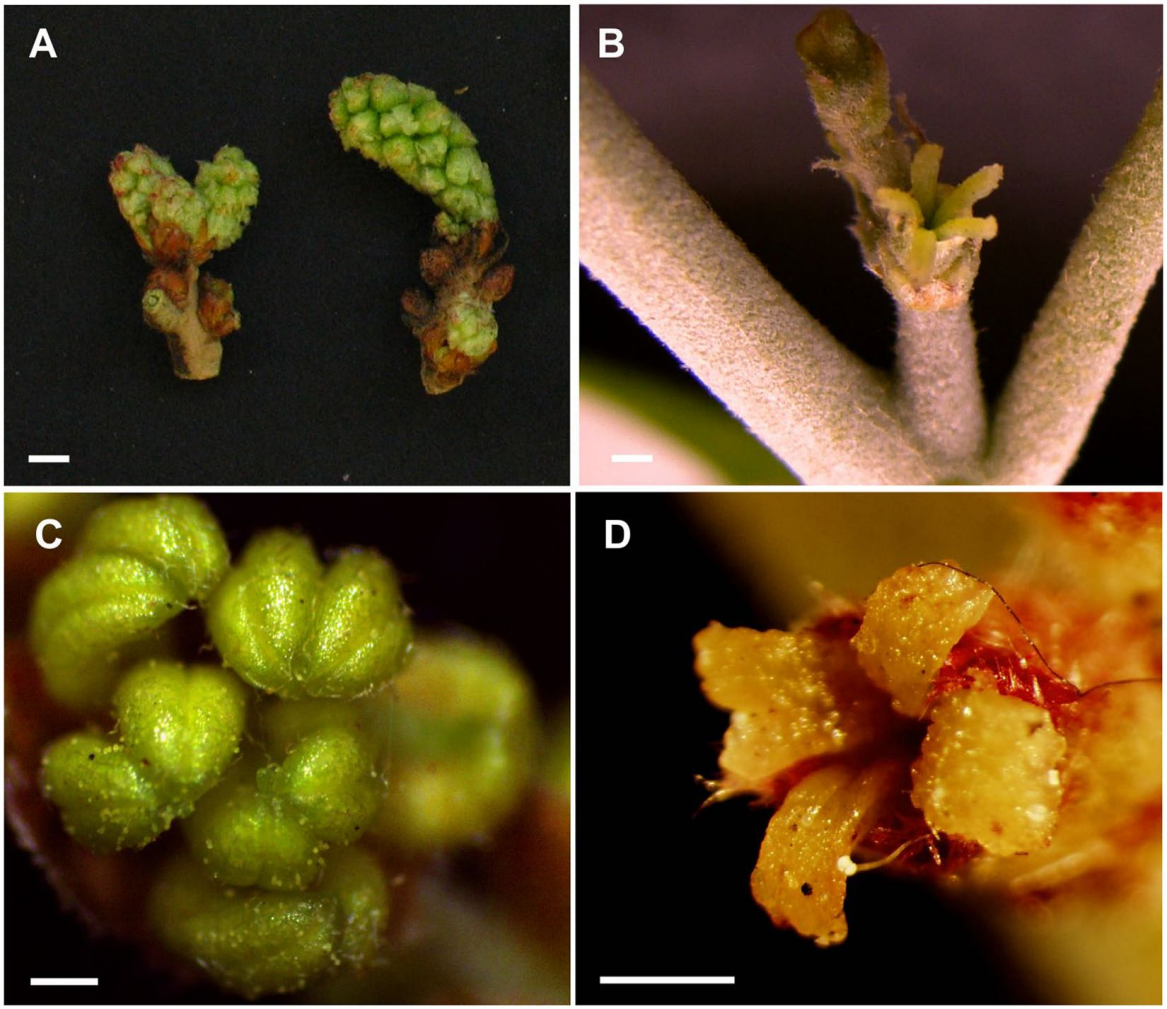
*Quercus suber* is a monoecious species with unisexual flowers at inception. (**A**) Male flowers developing in the previous season branches. (**B**) Female flower developing in the axil of a new leaf. (**C**) Anthers of a male flower. (**D**) Female unisexual flower with receptive stigmas (scale 1 mm).

*Q. suber* male and female flowers develop at different periods during the growing season. The male inflorescence, or catkin, develops in late winter, at the same time that winter-dormant buds break dormancy, containing ten up to twenty-five staminate flowers (Fig. 1A and C). The female inflorescences develop in mid spring, in the axils of newly formed leaves and contain up to eight carpellate flowers (Fig. 1B and D). Morphological observations suggested that neither the male nor the female flowers show aborted organs of the opposite sex.

The involvement of MADS-box genes during the development of unisexual flowers by inception has been reported in several species^7, 23, 27^. To address the importance of MADS-box genes in the reproductive identity of the *Q. suber* flowers, homologous genes were identified and their expression characterised during flower development. The highly-conserved MADS domain protein sequence of the Arabidopsis *PISTILLATA* was used as a query in a blast against the cork oak sequence database (www.corkoakdb.org) resulting in the identification of thirty-nine genes. These genes were then classified by phylogenetic inference using the Arabidopsis closest homologues available in the TAIR10 protein database (Figure S1).

The canonical MADS-box protein has four conserved regions: the M(ADS) domain, the I(ntervening) and K(eratin-like) regions and a variable C-terminal domain with conserved motifs^28^. MADS-box proteins can be divided into two sub-groups based on the length of the K domain: MIKC^c^ proteins have a shorter K domain whereas MIKC* have a longer one. According to the length of the predicted K domain, thirty-six proteins were classified as MIKC^c^ and three as MIKC*. The more conserved MIKC^c^ proteins were further divided into thirteen clades (GMM13, SQUA, AGL12, AGL17, AGL6, TM3, StMADS11, FLC, AGL15, AG, DEF/GLO and AGL2)^9^ (Figure S1).

**Figure 2:**
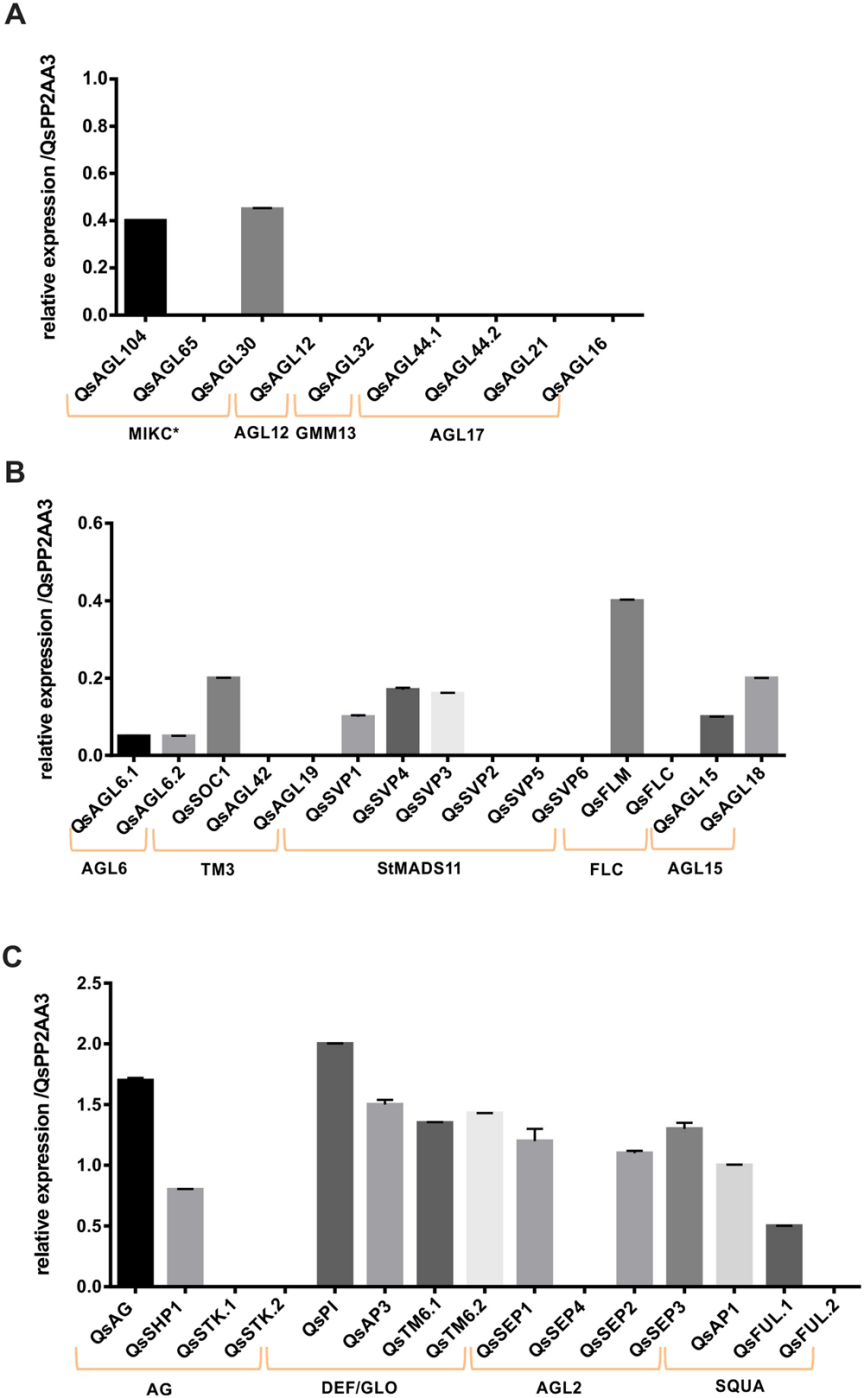
Expression of *Quercus suber* MADS-box genes in flowers. Gene expression analysis by RT-qPCR of *Quercus suber* MADS-box genes in a combined sample of male and female *Q. suber* flowers. (**A**) Genes associated to the MICK*, AGL12, GMM13, AGL17 clades. (**B**) Genes associated to the AGL6, TM3, StMADS11, FLC, AGL15 clades. (**C**) Genes associated to the AG, DEF/GLO, AGL2, SQUA clades. Error bars indicate standard deviation (s.d.) of three biological and technical replicates. QsPP2AA3 was used as reference gene.

Through phylogenetic inference it was possible to establish an association between the *Q. suber* MADS-box proteins and corresponding Arabidopsis proteins that are determinant factors in distinct plant organ developmental programs (leaf, root, shoot, flower and fruit organogenesis) (Figure S1). To evaluate which of the *Q. suber* MADS-box genes may have a role during flower development, a RT-qPCR was performed using a combined cDNA from male and female inflorescences at different developmental stages. No expression was detected for genes associated to the AGL12, GMM13 and AGL17 clades (Fig. 2A). The expression of genes of the AGL6, FLC, TM3, StMADS11, AGL15 and MIKC* clades was detected at lower levels (Fig. 2B). The highest level of gene expression was observed in *Q. suber* genes of the AG, AGL2, DEF/GLO and SQUA clades (Fig. 2C). These genes are homologous to Arabidopsis genes that have a pivotal role in floral organ identity.

### The ABCDE MADS-box transcription factors are conserved in *Q. suber*

**Figure 3:**
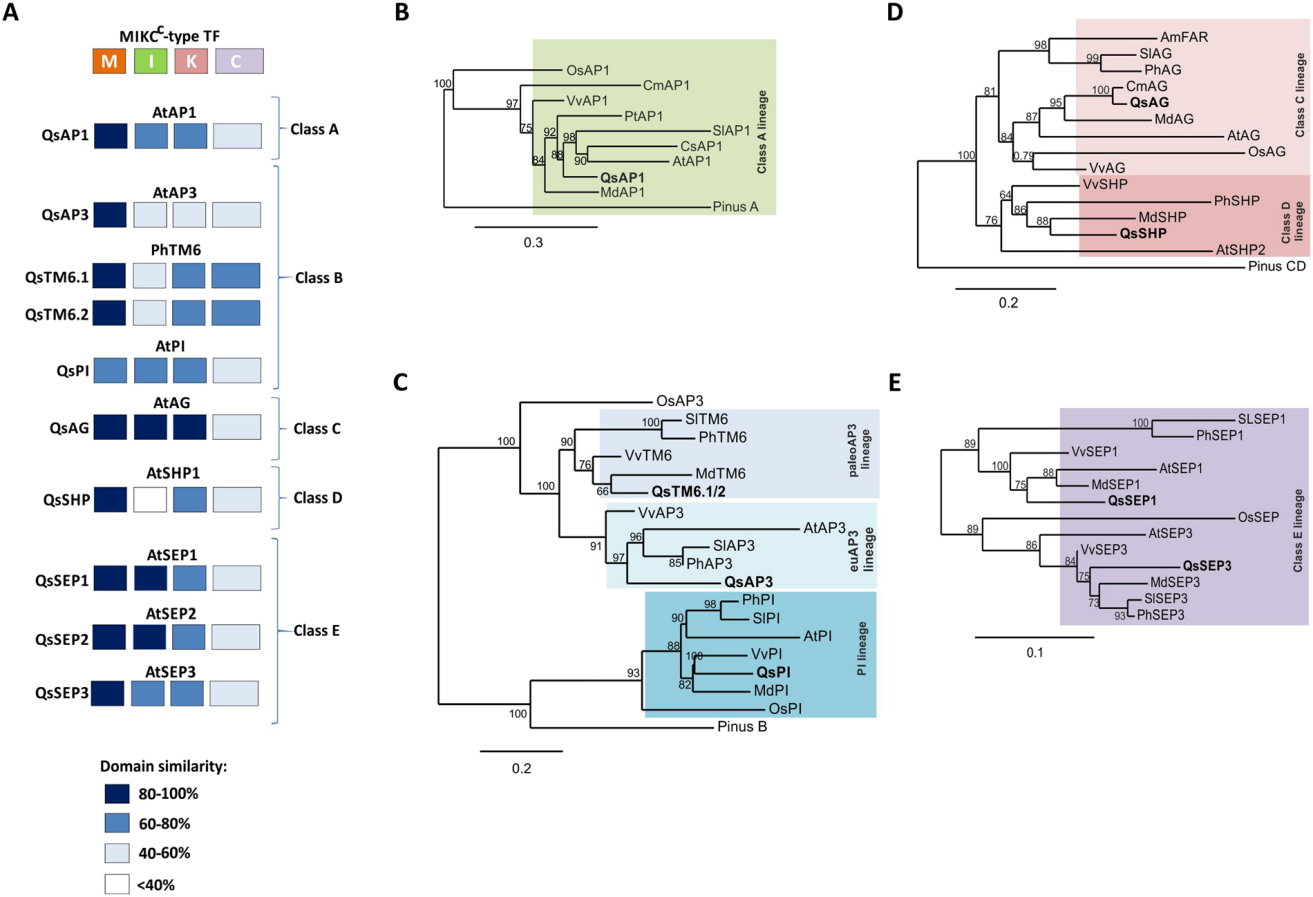
Phylogenetic profiling of the *Quercus suber* ABCDE homologous genes. (**A**) Domains similarity between *Q. suber* and *A. thaliana* (or *Petunia x hybrida* in the case of TM6) ABCDE MADS-box proteins were evaluated by residue identity in the four conserved domains (M - MADS; I - Intervening region; K - Keratin-like domain; C - C-terminal domain). (**B**) Phylogenetic analysis of the SQUA family. (**C**) Phylogenetic analysis of the DEF/GLO family. (**D**) Phylogenetic analysis of the AG family. (**E**) Phylogenetic analysis of the AGL2 family. *Q. suber* proteins marked in bold. Independent lineages are distinguishable by different colours (Green – A-class lineage; light purple – paleoAP3 lineage; light blue – euAP3 lineage; dark blue – PI lineage; light pink – C-class lineage; dark pink – D-class lineage; dark purple – E-class lineage).

The phylogeny of the *Q. suber* ABCDE-like genes was further analysed using homologues of distinct angiosperm species and, when available, a homologue from a gymnosperm representative species (Fig. 3). In the A-class lineage, QsAPETALA1 (QsAP1) grouped closely with AP1-like proteins from other perennial tree species, particularly with the one from *Castanea mollissima*, a species phylogenetically close to *Q. suber* (Fig. 3B). By comparing the protein domain structure and amino acid similarity within each domain of the *Q. suber* and the Arabidopsis homologue it is predicted that QsAP1 has the four canonical domains and that the level of conservation is very high, particularly in the MADS domain (above 80%) (Fig. 3A).

The B-class clade can be divided into three independent lineages: PI, euAP3 and paleoAP3^29, 30^. Within the PI lineage, *QsPISTILLATA* was grouped closer to other PI-like proteins (Fig. 3C). QsPI four putative conserved regions are 40–80% identical to the AtPI counterparts (Fig. 3A). Despite a lower level of residue identity (particularly in the MADS domain), QsPI has a completely conserved PI motif (short amino acid sequence that characterises the PI lineage and is essential for protein function^29^) in the C-terminal region (Figure S2A).

The paleoAP3 and euAP3 lineages diverged after duplication at the base of the core eudicots and the major difference between lineages is a specific motif in the C-terminal domain^29^. One APETALA3 (AtAP3) homologue (QsAP3) was identified in the euAP3 lineage (Fig. 3C). QsAP3 has a partially conserved euAP3 motif (Figure S2A) and, with the exception of the MADS domain, shows a low degree of residue conservation (less than 60%) when compared to AtAP3, which suggests a possible functional divergence (Fig. 3A). Two paleoAP3 *Q. suber* proteins (QsTM6.1 and QsTM6.2) were also identified (Fig. 3C). QsTM6.1 differs from QsTM6.2 by having fourteen extra aminoacids in the I-region. Both QsTM6.1 and QsTM6.2 protein domains are very similar to the *Petunia x hybrida* TM6-like (PhTM6 was used because there is no TM6 homologue in Arabidopsis) (Fig. 3A), however the paleoAP3 motif is not completely conserved (Figure S2A).

The phylogenetic analysis of the AG clade resulted in the grouping of one *Q. suber* protein with the Arabidopsis D-class protein SHATTERPROOF1 (AtSHP) and three *Q. suber* proteins with the Arabidopsis D-class protein SEEDSTICK (AtSTK) (Figure S1). Two of the three *STK-like Q. suber* genes were not expressed in flowers (Fig. 2C). A detailed analysis on the conserved AG motifs (motif I and motif II) in the C-terminal domain^31, 32^ suggested that QsAG (the putative *STK*-like gene that is expressed in flowers) has higher proximity to AtAGAMOUS (the canonical C-class protein) than to AtSTK. On the contrary, the AG motifs I and II of QsSHP were more similar to the ones of AtSHP (Figure S2B). A new phylogenetic analysis was performed using AG-like and SHP-like proteins from other species and resulted in the placement of QsAG together with other AG-like proteins (Fig. 3D). QsSHP was placed in the same clade of AtSHP (Fig. 3D), however, a lack of residue conservation, particularly in the I region (Fig. 3A), and a complete deletion of the AG motif II (Figure S2B) may suggest a divergent role for QsSHP during *Q. suber* flower development.

The E-class includes the *SEPALLATA*-like genes, which are unique to angiosperms. Four genes were identified in the cork oak database (Figure S1) but *QsSEP4* was not expressed in flowers (Fig. 2C). The E-class clade was divided into two different lineages, the SEPALLATA3 (SEP3) (containing one *Q. suber* protein), and the SEP1/2 lineage (containing two *Q. suber* proteins, QsSEP1 and QsSEP2) (Fig. 3E). Of the two SEP motifs that characterise the SEP1/2 lineage, only SEP motif II was conserved in both QsSEP1 and QsSEP2 (Figure S2C). QsSEP3 had partially conserved SEP motifs (I and II), which are characteristic of the SEP3 lineage (Figure S2C).

### Several *Q. suber* ABCDE-like genes expression is sex-biased

**Figure 4:**
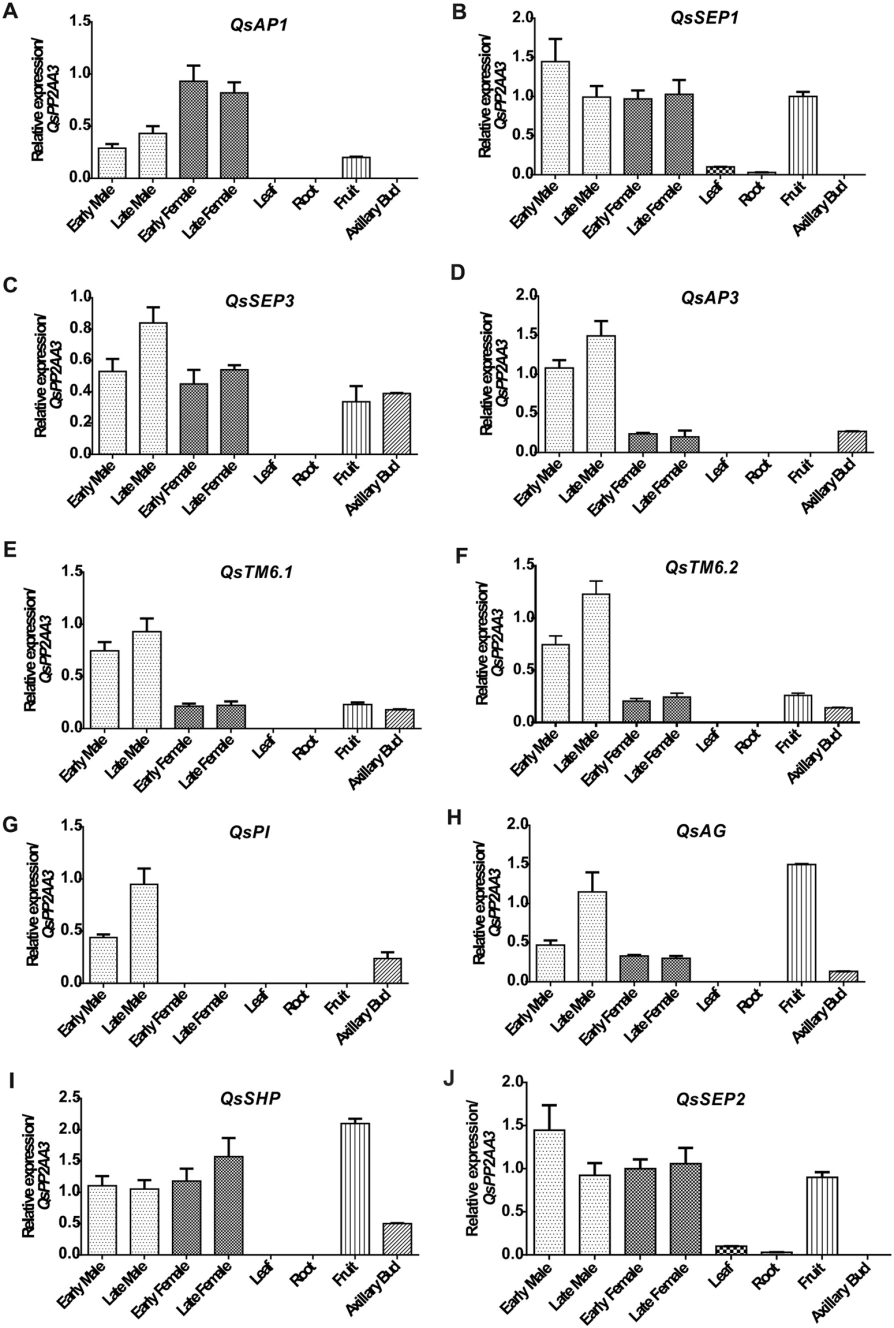
Some *Quercus suber* ABCDE gene expression is sex-biased. Gene expression analysis by RT-qPCR of *Q. suber* ABCDE homologue genes was measured in different tissues: early and late stages of male or female flower development, axillary bud, fruit, leaf, and root. (**A**) *QsAP1*; (**B**) *QsSEP1*; (**C**) *QsSEP3*; (**D**) *QsAP3*; (**E**) *QsTM6.1*; (**F**) *QsTM6.2*; (**G**) *QsPI*; (**H**) *QsAG*; (**I**) *QsSHP* and (**J**) *QsSEP2*. Error bars indicate standard deviation (s.d.) of three biological and technical replicates. *QsPP2AA3* was used as reference gene.

During flower development, a tight spatiotemporal regulation of MADS-box gene expression is necessary for the development of fully functional floral organs. To determine the expression patterns of *Q. suber* ABCDE-like genes, a RT-qPCR analysis was performed using two pools of cDNA samples from both male and female flowers, at different developmental stages. One pool contained early flower developmental stages, from early onset to pre-maturation. The other pool contained flowers in late stages of flower development, from post-maturation to pollen shedding (in male flowers), or to early stages of fruit development (in female flowers). *Q. suber* ABCDE-like genes were predominantly expressed in the flowers, when compared to expression in other tissues tested (leaf, bud, root and fruit). Most of these genes were also significantly expressed in the fruit and some in the axillary bud (Fig. 4).

*QsSEP1*, *QsSEP2* and *QsSEP3* were expressed at a similar level in early and late stages of male and female flower development. *QsSEP1*, *QsSEP2* and *QsSEP3* were expressed in the fruit but only *QsSEP3* had expression in the axillary bud (Fig. 4B,C and J). *QsSEP1* and *QsSEP2* were the only *Q. suber* ABCDE-like genes expressed in leaves or roots. *QsSHP* was expressed at the same level in both male developmental stages and in the early female pool but its expression increased significantly in late stages of female flower development and in the fruit (Fig. 4I). Only *QsAP1* was significantly more expressed in the female than in the male flowers, particularly in early stages of female flower development (Fig. 4A). *QsAP3, QsTM6.1, QsTM6.2, QsPI* and *QsAG* had higher expression in male flowers than in female, particularly in post-maturation stages. These five genes were also expressed in axillary buds (Fig. 4D–H). Similarly to *QsSHP*, *QsAG* was highly expressed in the fruit (Fig. 4H and I). *QsPI* is the only gene that was uniquely expressed in the axillary buds and male flowers, particularly in late developmental stages, not being detected in female flowers (Fig. 4G). Expression analysis showed that *Q. suber* ABCDE-like genes are differentially regulated in male and female flowers suggesting an organ-specific gene regulation.

### Some *Q. suber* ABCDE-like genes have a function in flower induction and organ identity

**Figure 5:**
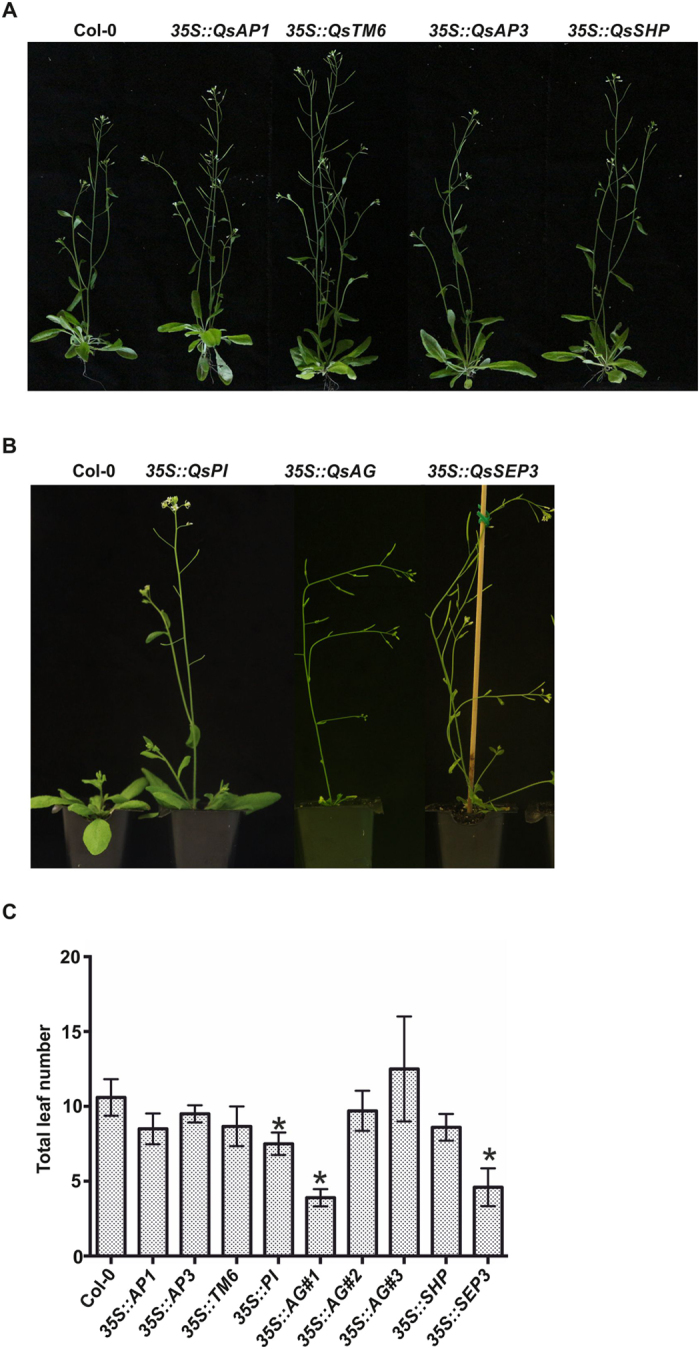
Overexpression of *Quercus suber* ABCDE homologues genes in *A. thaliana* generate distinct plant phenotypes. (**A**) From left to right: wild-type (Col-0), *35 S::QsAP1, 35 S::QsTM6, 35 S::QsAP3* and *35S::QsSHP* plants. (**B**) From left to right: wild-type (Col-0), *35S::QsPI, 35S::QsAG* and *35S::QsSEP3* plants. (**C**) graphic display of the total number of leaves of *A. thaliana* WT and overexpression plants during flowering. Error bars indicate standard deviation (s.d.). Asterisks indicate p-values ≤ 0.05 determined after performing a Students t-test.

**Figure 6:**
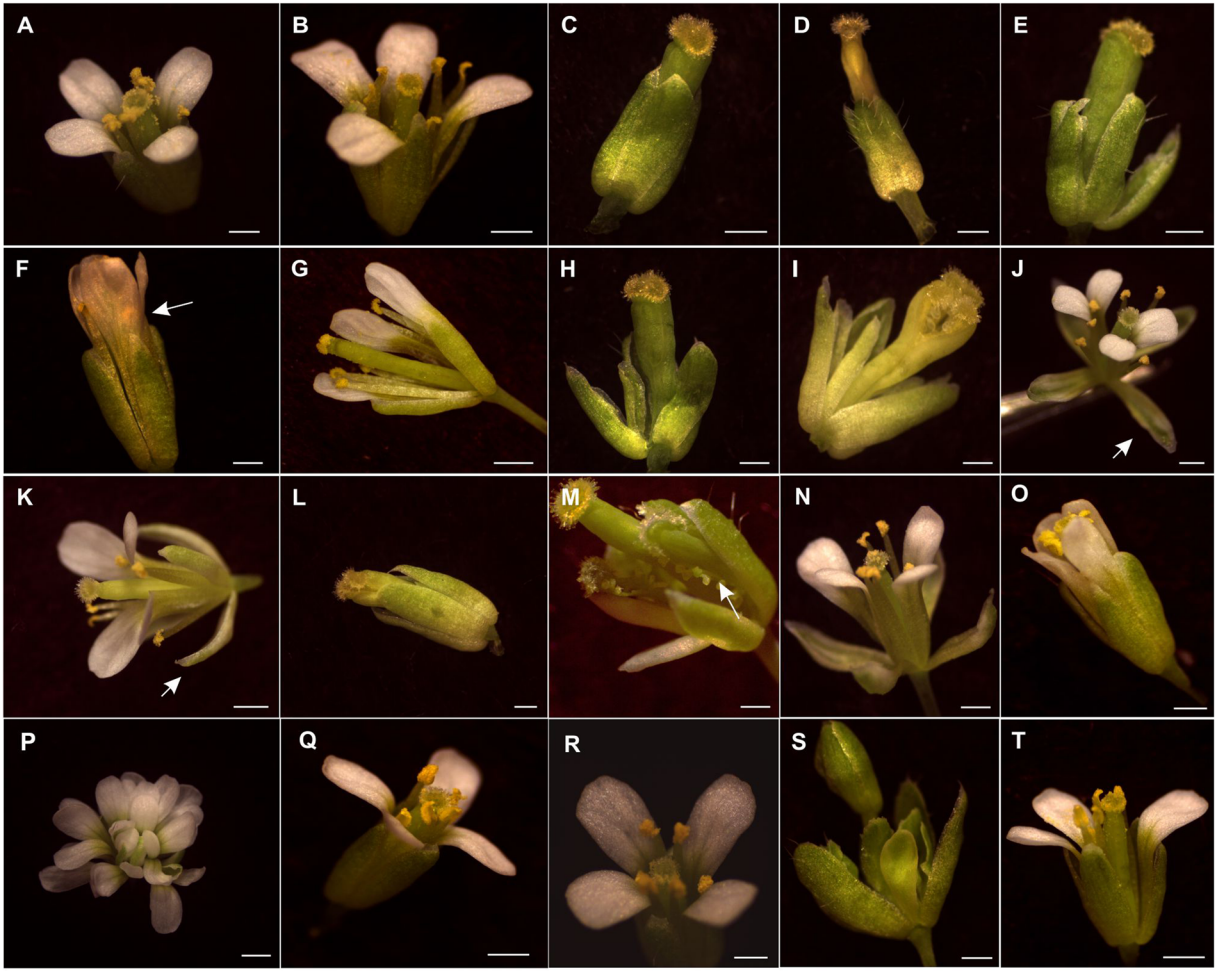
*A. thaliana* plants overexpressing *Q. suber* ABCDE genes display distinct flower phenotypes. (**A**) Wild type (Col-0). (**B**) *35S::QsAP3*. (**C**) *ap3-3* mutant. (**D**) *35S::QsAP3* in the *ap3-3* background. (**E**) *pAtAP3::QsAP3* in the *ap3-3* background. (**F**) *35S::QsTM6* (arrow represent the lack of petal curvature). (**G**) *35S::QsTM6* flower without a sepal and petal. (**H**) *pAtAP3::QsTM6* in the *ap3-3* background. (**I**) *35S::QsTM6* in the *ap3-3* background. (**J**) *35S::QsPI* (arrow represent a sepaloid petal). (**K**) *pAtPI::QsPI* (arrow represent a sepaloid petal). (**L**) *pi-1* mutant. (**M**) *pAtPI::QsPI* in the *pi-1* background (arrow represent ectopic ovules). (**N**) *35S::QsPI* in the *pi-1* background. (**O**) *35S::QsAG*. (**P)** *ag-1* mutant. (**Q**) *35S::QsSHP*. (**R**) *35S::QsSEP3*. (**S**) *sep1 sep2 sep3* mutant. (**T**) *35 S::QsSEP3* in the *sep1 sep2 sep3* background. Scale bar: 0.5 mm.

To analyse a potential conservation in the function of cork oak ABCDE-like genes, Arabidopsis transgenic lines overexpressing their coding regions were obtained. *QsAP1* and *QsAP3* overexpressing plants were similar to the wild type (WT) (Fig. 5A and C). To evaluate whether the absence of phenotypic differences could be related to down-regulation of the transgenes, an RT-qPCR using RNA from floral tissues was performed. *QsAP1* was highly expressed in *QsAP1* transgenic plants, but the *QsAP3* transgene was weakly expressed in the *QsAP3* overexpressing plants (Figure S3). Flowers from plants overexpressing *QsAP3* were similar to WT, with no organ homeotic conversion (Fig. 6A and B). To assess whether *QsAP3* was able to restore the floral defects of the *ap3-3* (mutant lacking petals and stamens) (Fig. 6C), mutant plants were complemented with *QsAP3* coding region driven by the 35 S promoter or by a 0.5Kbp fragment of the *AtAP3* promoter. *QsAP3* driven by the *AtAP3* native promoter was expressed significantly more that when driven by the 35 S promoter (Figure S3), however, in both strategies, expression of *QsAP3* in the *ap3-3* background failed to rescue the development of petals and stamens (Fig. 6D and E).

*QsTM6.1* overexpressing lines showed mild differences when compared to WT, displaying slight reduced shoot apical dominance (Fig. 5A) and petals that did not curve outwards (Fig. 6F), with no other observed flower defects (Fig. 6G). *QsTM6.1* expression failed to complement the floral defects of *ap3-3* when driven from the *AtAP3* native promoter (Fig. 6H). When driven by the 35 S promoter *QsTM6.1* rescued the fertility in 15% of the *ap3-3* flowers, despite the majority of the flowers being similar to the mutant *ap3-3* (Fig. 6I). Plants overexpressing *QsPI* flowered significant early under long days when compared to WT plants (Fig. 5B and C) and the flowers displayed homeotic conversions of sepals into petaloid structures (Fig. 6J). Petal cells of the *35::QsPI* flowers were similar to the WT (Figure S5B, supporting method) but the flanks of the sepal adaxial epidermis contained typical petal conical cells (Figure S5C), suggesting that petal identity has expanded to the sepal whorl. No alterations were found in the pistils of *QsPI* overexpressing plants. Complementation of the *A. thaliana pi-1* mutant phenotype (mutant flower lacking petals and stamens) (Fig. 6L) was tested using a *QsPI* coding region controlled by a 1.5 kb fragment of the native Arabidopsis promoter (*pPI::QsPI*). *pPI::QsPI* transgenic plants had the same flower defects as the overexpressing line (Fig. 6K). Flowers of *pPI::QsPI* transgenic plants in the *pi-1* background had restored petals and stamen-like structures with ectopic ovules (Fig. 6M). Some of the flowers of *pPI::QsPI* in the *pi-1* background plants had fully functional stamens that enabled self-fertilisation. Complete rescue of stamens was achieved by complementing *pi-1* with *QsPI* controlled by the 35S promoter (Fig. 6N).

Phenotype analysis of *QsAG* overexpressing plants revealed a variety of phenotypes that could be sorted into three groups. The first included plants with early flowering, early stem termination and small curly leaves (Fig. 5C, *35 S::AG#1* and Figure S4); the second group contained plants with reduced shoot apical dominance and altered phyllotaxy, but no early flowering (Fig. 5C, *35 S::AG#2*); and finally a third group of plants that were very similar to the WT (Fig. 5C, 35 S::AG#3). RT-qPCR analysis showed that the *QsAG* transgene is significantly more expressed in plants of the first and second groups (Figure S3). None of the independent transgenic plants had flowers with homeotic conversion of sepals and petals into carpels and stamens, respectively (Fig. 6O). *QsSHP* overexpressing plants had slightly early flowering (Fig. 5A and C), but similarly to the *35 S::QsAG* plants, there was no organ defect including no conversion of the perianth organs into carpels or stamens (Fig. 6Q). *QsAG* or *QsSHP* coding regions driven by the 35S promoter were not able to rescue the defects of the *ag-1* mutant (Fig. 6P).

Plants overexpressing *QsSEP3* had an extremely early flowering phenotype (Fig. 5B and C). *QsSEP3* flowers did not show any kind of homeotic transformation of flower organs (Fig. 6R) but expression of *QsSEP3* in the *sep1 sep2 sep3* background (mutant containing predominantly sepals, Fig. 6S) restored completely the fertility of the mutant (Fig. 6T).

### Different protein-protein interactions might control different aspects of *Q. suber* flower development

**Figure 7:**
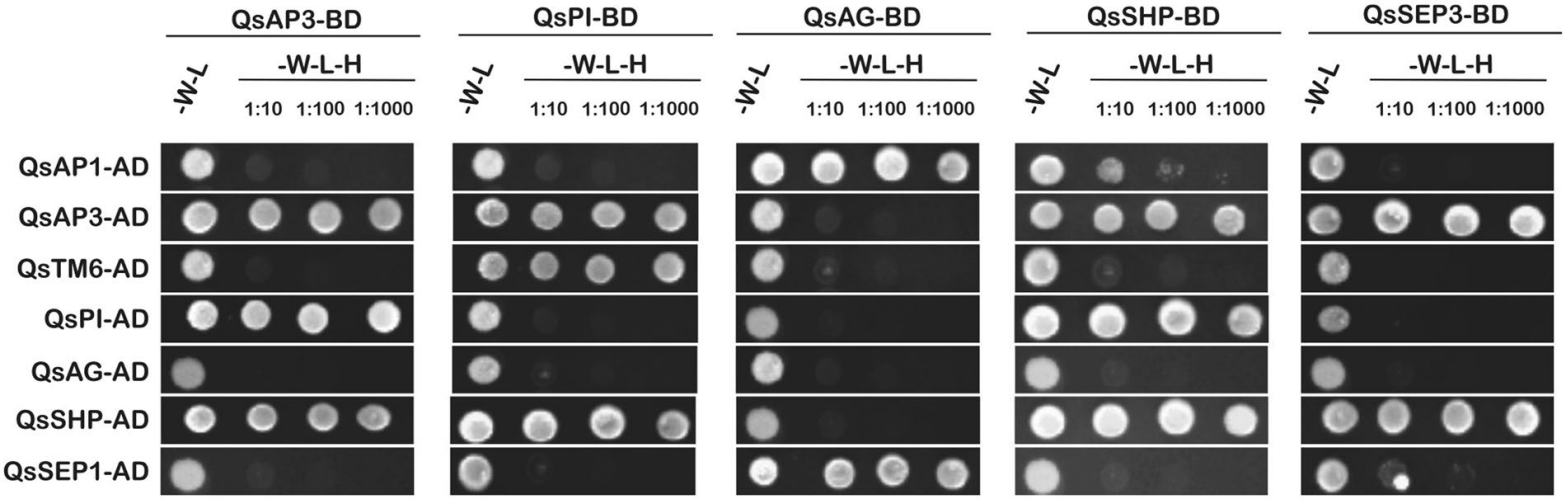
The combinatorial activity of *Q. suber* ABCDE-like proteins is partially conserved. Different interaction combinations between *Q. suber* ABCDE-like proteins were tested. *Q. suber* ABCDE-like proteins were fused to either GAD4-activation domain (AD) or to GAL4-binding domain (BD). Double yeast transformations were first tested for plasmid presence by growing cells in Synthetic Defined (SD) medium without tryptophan and leucine (-W-L). Ability to interact was evaluated in SD medium without tryptophan, leucine and histidine (-W-L-H). Protein Interaction strength was assessed by using successive cell culture dilutions (1:10, 1:100 and 1:1000). Each cropped image corresponds to a matching single plate in which the yeast double transformants were selected.

The ability to bind DNA in higher-order complexes is the cornerstone of the MADS-box protein function and determines the identity of flower organs in several species^33^. Stamen development is dependent on the formation of quaternary complexes of B-, C- and E-class MADS-box proteins, whereas carpels depend on the combinatorial action of C- and E-class MADS-box proteins. Thus, a change on the ability to establish protein-protein interactions could be part of the developmental process that may be involved during unisexual flower formation. The ability of *Q. suber* ABCDE-like proteins to dimerise was evaluated in a yeast two-hybrid (Y2H) experiment by fusing the coding regions of the *Q. suber* ABCDE-like genes to the activation or binding domain of the *GAL4* transcription factor. QsPI was able to interact with QsAP3 and QsTM6. QsAP3 self-dimerised but did not interact with its closest relative, QsTM6 (Fig. 7, first two columns). Interestingly, QsAP3 and QsPI (B-class proteins) interacted with QsSHP (C-class) but not with QsAG (C-class), suggesting that QsSHP might have retained the C-class function in the formation of higher order complexes in the reproductive whorls (Fig. 7). Furthermore, QsAP3 and QsSHP formed a complex with QsSEP3 (Fig. 7). No interaction was observed between QsAG and QsSEP3, or with any B-class proteins. QsAG only interacted with QsAP1 and QsSEP1. QsSEP1 or QsAP1 fused to the binding domain of GAL4 were not used in this Y2H assay because both proteins were able to activate transcription, as seen in other species^34, 35^.

## Discussion

Unisexuality by inception likely derives from failure to initiate organ primordia^36^. Species with this trait represent an excellent opportunity to study the regulatory mechanisms that control the early establishment of male and female flower organ identity. For the past decades, unisexuality by inception has been studied in dioecious species pointing to ABCDE-like genes as one of the molecular switches controlling the development of male or female flowers^5–7, 23^. Differential regulation of ABCDE-like genes involved during the formation of unisexual flowers by inception in dioecious species may be conserved in species with a similar trait but with different sexual strategies (e.g. monoecious), but no such information is presently available. The RNAseq study performed by Rocheta and colleagues (2014) points to several MADS-box genes as being deregulated in male and female libraries of *Q. suber* but a role for these genes in floral organ identity is still unknown. To bridge this gap, a study was conducted to infer on the role of ABCDE-like genes in the early establishment of the unisexual flowers by inception of *Q. suber*.

Following the reasoning of the canonical ABCDE model, early establishment of the *Q. suber* female flower identity should depend on the activity of C- and E-class genes and the male flower identity on C-, B- and E-class genes^37, 38^. The E-class genes function as co-factors for the establishment of all the flower organs as suggested by the *sep1 sep2 sep3 sep4* mutant that develops leaves instead of flowers^14, 39^. In *Q. suber*, the expression profile of *QsSEP1, QsSEP2* is identical and both genes are expressed during male and female flower development. *QsSEP3* is equally expressed in female flowers but is significantly more expressed in late stages of male flower development, suggesting a role in pollen maturation.

Arabidopsis plants overexpressing *QsSEP3* display early flowering but show no floral defects, a result consistent with the function of *SEP3*-like genes in other species^34, 40, 41^. However, some reports have suggested different roles for *SEP3*-like genes in floral organ identity. Arabidopsis plants overexpressing *AtSEP3* show homeotic transformation of sepals into carpeloid structures with ectopic ovules in the external surface, suggestive of *AtAG* ectopic activation in the sepal whorl^42^, whereas the overexpression of the lilium *SEP3*-like creates indeterminate flowers in Arabidopsis invocative of a compromised C- function^43^. Thus, the *SEP3*-like gene function in floral transition is likely conserved but not the ability to promote floral homeotic changes, which may be species-specific and correlated to a divergent evolution of DNA-binding activity of downstream targets or interacting partners.

In the stamen and carpel whorls of Arabidopsis and petunia, interaction of SEP3-like with C-class proteins and with the AP3-like/PI-like heterodimers is necessary to proper organ development^35, 44–46^. In *Q. suber*, E-class proteins might be fulfilling their role in organ identity because they interact with B- and C-class proteins. Furthermore, it is likely that QsSEP3 is able to interact with native Arabidopsis B-class and C-class proteins because *QsSEP3* overexpression rescues the sterile phenotype of the triple *sep1 sep2 sep3* Arabidopsis mutant.

**Figure 8:**
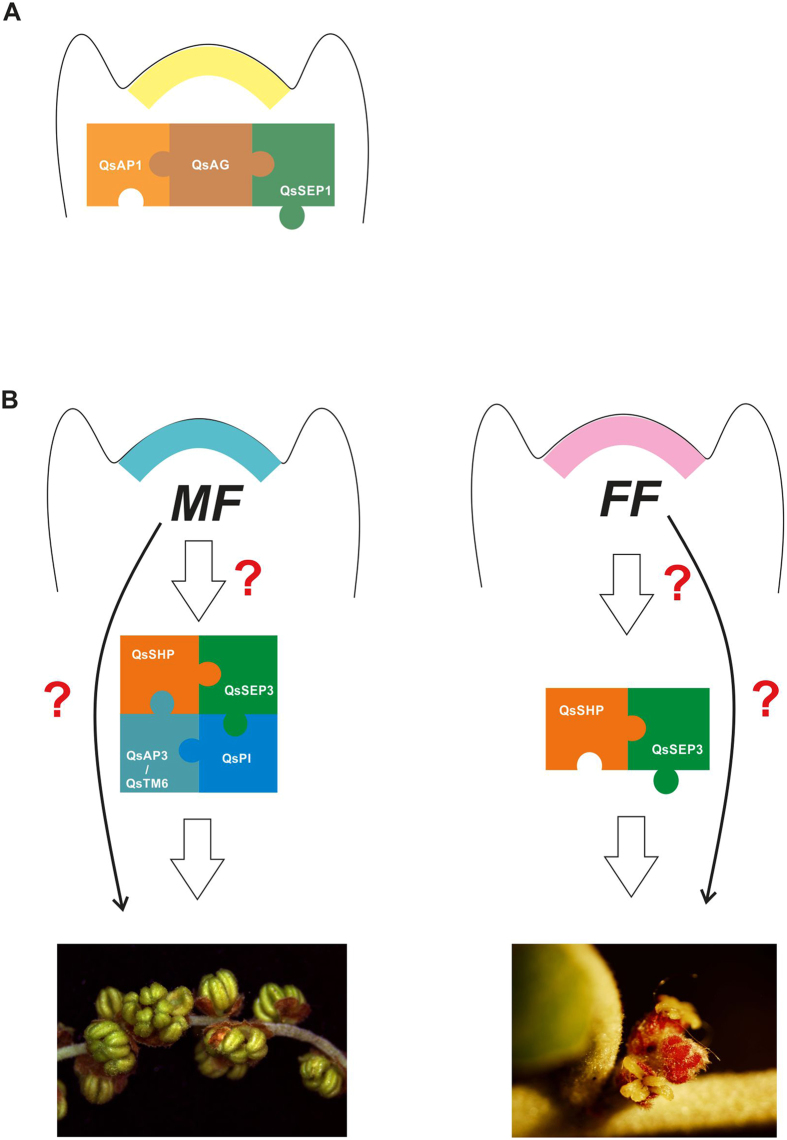
Model on the putative role of MADS-box genes in the reproductive development of *Q. suber*. (**A**) The interaction between QsAG/QsAP1/QsSEP1 could be related to the transition from vegetative to the reproductive development. (**B**) The QsSHP/QsAP3/QsSEP3/QsTM6/QsPI combinatorial complex is involved in male flowering, whereas the QsSHP/QsSEP3 complex is involved in female flowering. Regarding this hypothesis, unisexual flowering in *Q. suber* could be determined by a MF (male factor) or an FF (female factor) that may control the MADS genes. However, it is possible that an MF or FF could determine sex identity in parallel or independently from the MADS-box genes.

There has been some studies reporting interaction between AP1-like and SEP-like proteins in Arabidopsis and rice^34, 47^, and an Y3H experiment performed in Arabidopsis has shown that AtAP1, AtSEP3 and SEUSS form a higher-order complex that act as repressor of *AtAG* in sterile floral organs^48^. In *Q. suber*, QsSEP1 interacts with QsAG. Interestingly, QsAP1 also interacts with QsAG, which has not been previously reported in any other species. Assuming that QsAP1, QsAG and QsSEP1 are able to interact *in vivo*, the establishment of a QsAP1-QsAG-QsSEP1 protein complex may be related to an undisclosed mechanism that controls floral meristem development (Fig. 8).

The function of C-class genes in reproductive organs is tightly conserved in angiosperms, and with the notable exception of a basal angiosperm (Illicium)^49^, in which a C-class gene is expressed in tepals, no other example has been described of a naturally occurring C-class gene expressed in non-reproductive organs. The Arabidopsis C-class gene, *AtAG*, controls the identity of stamens and carpels, but also the determinacy of the flower meristem^50, 51^. Contrarily to what happens in Arabidopsis, the majority of the angiosperm species has two or more *AG*-like genes, and in some species, like maize or rice, different C-class genes share the canonical C-class functions^52, 53^. Two putative *AG*-like genes are expressed in the *Q. suber* flowers, one that clusters with *AtAG* and the other with *AtSHP1* (which has a canonical role in ovule identity^15^). In *Q. suber*, the embryo sac development is delayed^3^, occurring weeks after pollination. Therefore, expression of *QsSHP* in both male and female flowers is not likely to be associated with ovule development and it is likely that *QsAG* and *QsSHP* are both C-class genes.

Arabidopsis plants overexpressing *QsAG* display severe phenotypic defects such as early flowering and early development arrest. These phenotypes were also observed in plants overexpressing *AG*-like genes from other species^51, 54–56^. Some of the plants overexpressing *QsAG* display curly leaves, also observed in *35 S::AtAG* plants, a phenotype associated with *curly leaf*, a mutant in a gene that epigenetically blocks *AtAG* expression in WT Arabidopsis leaves^57^. This suggests that *QsAG* might have retained some of the canonical C-class gene functions. No phenotypic alterations were observed in *35* *S::QsSHP* plants. Arabidopsis flowers overexpressing *QsAG* and *QsSHP* do not display homeotic conversions of petals into stamens and sepals into carpels, as the ones observed in *35S::AtAG* Arabidopsis plants or in plants overexpressing *AG*-like genes of petunia, cucumber, rice or lilium^51, 54–56^. The lack of flower homeotic conversions and the failure to rescue the *ag-1* mutant suggests a certain degree of functional divergence. One possibility to explain the lack of expected phenotypes is that both *QsAG* and *QsSHP* may be needed to fulfil a complete C-class function as often observed in many core eudicots^52, 58, 59^, which is supported from the observation that both *QsAG* and *QsSHP* have a similar expression pattern in early stages of the *Q. suber* male and female flower development.

The role of QsSHP and QsAG in *Q. suber* flower organ identity may be conserved, as they are able to interact with an E-class protein. Unexpectedly, QsSHP also interacts with QsAP3, a B-class protein. The interaction of B-class proteins with C-class proteins without the mediation of an E-like protein has not been previously reported in eudicots. Examples of such interaction have only been shown in gymnosperms, where B- and C-like proteins interact directly to control male cone development^60^, and in some basal angiosperms species^61^. The almost complete deletion of the AG motif II from QsSHP protein may allow the interaction of with unusual partners, such as B-class proteins. Direct interaction between B- and C-class proteins may pinpoint the particular nature of the protein complexes that are established during floral organ primordia development in *Q. suber*.

In *Q. suber*, four B-class genes were identified based on lineage specific motifs (*QsAP3*, *QsPI*, *QsTM6.1* and *QsTM6.2*). The expression profile of the *AP3*-like and *TM6*-like genes in flowers is very similar, with significantly higher expression in male flowers, and at a lower level in female flowers. *AP3*-like genes are usually expressed in the petal and stamen whorls but there are examples of *AP3*-like genes also weakly expressed in the carpel whorl^62^. *TM6*-like genes are usually expressed in petals, stamens and carpels in early stages of flower development and in ovules and transmitting tissue in later stages^63, 64^, although its function has been shown to be restricted to stamen development^65^. In *Q. suber*, the overexpression of *QsAP3* failed to rescue petal and stamen development of the *ap3-3* mutant or promote homeotic conversion of carpels into stamens, opposite to what happens in *35S::AtAP3* flowers^66^, suggesting a biochemical divergence within the euAP3 lineage. However, QsAP3 is still able to take part in higher order protein complexes with other *Q. suber* B- and E-class proteins (as well as with C-class proteins). Similarly to *QsAP3*, overexpressing the *Q. suber TM6*-like (*QsTM6.1*) in *A. thaliana* failed to promote homeotic conversion of carpels into stamens but was able to partial recover the stamens of the *ap3-3* mutant, suggesting that *QsTM6.1* may have retained some of the paleoAP3 canonical function.

Obligatory heterodimerisation of AP3- and PI-like proteins controlling petal and stamen development is preserved across the angiosperms^67, 68^ and is also needed to specify male cones in gymnosperms^60^. In *Q. suber*, QsPI interacts with both QsAP3 and QsTM6 suggesting that the obligatory interaction may be preserved in this species. Arabidopsis plants overexpressing *QsPI* show flowers with homeotic conversion of sepals into a petaloid organ, a phenotype that has been previously described in Arabidopsis plants overexpressing *AtPI* ^69^. Furthermore, *QsPI* expression was able to rescue petal and stamen development in the *pi-1* background suggesting a conserved function in organ identity. As expected, no homeotic conversion of carpels into stamens was observed and this may be due to the lack of an *AP3*-like interactor expressing in the fourth whorl of the *A. thaliana* wild-type flower, suggesting that *QsPI* needs to heterodimerise to retain function.

*QsPI* expression is restricted to the *Q. suber* male flowers. Lack of *QsPI* expression in *Q. suber* female flowers may be tightly interconnected with the lack of male organs in these flowers, as it has been suggested in other unisexual species, where B-class genes need to be transcriptionally repressed to allow the development of female organs^5, 7, 27^. In *Q. suber*, obligatory interaction of B-class proteins and establishment of a complex with C- and E-class proteins might be needed in the male flowers (Fig. 8) promoting stamen development and/or inhibiting carpel primordia, similarly to what happens in Arabidopsis when the ectopic expression of *AtPI* and *AtAP3* in the fourth whorl blocks carpel development^66^. The lack of *QsPI* expression in female flowers may allow the interaction of C- and E-class proteins to promote carpel development (Fig. 8) not allowing stamen primordia to be initiated. Considering this hypothesis, the mechanisms that establish unisexual flowers in *Q. suber* might be acting upstream of the regulatory module of the MADS protein complexes, controlling the regulation of *QsPI* expression. The temporal regulation of *QsPI* may limit the deployment of a homeotic change to a specific time frame (during male flowering) without causing potentially deleterious effects such as transformation of the carpel into stamens in a later reproductive phase (during female flowering). In the dioecious *S. oleracea*, there is genetic evidence that regulation of B-class genes might have a direct role in flower organ identity/unisexuality, which might imply that regulation of unisexuality by inception might be conserved. Several questions remain open, in particular the identification of the sex determination factors that may be acting upstream of the MADS-related pathway or in a parallel/independent-signalling pathway (Fig. 8).

The elucidation of the mechanism responsible for the establishment of unisexual flowers has an evolutionary, ecological and economic importance. Although in the past decades several genetic studies have provided a framework to partially explain sex-determining pathways, a common mechanism controlling the onset of male and female flowers at inception is still illusive. Here, we propose a simple model for the regulation of flower organ identity in the monoecious species *Q. suber*, via the modulation of MADS-box transcription factor function. Taking into consideration the similar nature of flower development of other tree species, it is likely that this model for organ identity may be conserved.

## Experimental procedures

### Plant material

*Arabidopsis thaliana* ecotype Columbia (Col-0) and mutant seeds were acquired from the Nottingham Arabidopsis Stock Centre. Seeds were sown in Murashige and Skoog agar medium and incubated under long day conditions (16 hours light/8 hours dark) at 20 °C in controlled environmental growth rooms, with light intensity of 30 μE m^−2^ s^−1^. Approximately 10 days after sowing, plantlets were transferred to pots containing a 4:1 (v:v) mixture of turf rich soil and vermiculite. Overexpression constructs were introduced into *A. thaliana* ecotype Col-0 using the GV3101 agrobacterium strain and the floral dip method^70^. Transgenic plants were selected in medium supplemented with hygromycin. After checking for single insertion lines, four independent lines were selected from each construct. Functional studies were conducted using plants homozygous for the transgene. *Q. suber* plant organ samples (root, leaf, flowers, fruit and bud) were collected from three *Quercus suber* trees located in the Minho University campus. Early and late stages of male and female flower development were selected based on the phenological stages defined by Varela and Valdiviesso (1996).

### Phylogenetic methods

The cork oak MADS-box DNA sequences were obtained by performing a BLAST in the cork oak database (http://corkoakdb.org) using the *Arabidopsis thaliana PISTILLATA* protein sequence as a query. The cork oak MADS-box homologous protein and DNA sequences were obtained by performing a BLAST at the NCBI database (http://www.ncbi.nlm.nih.gov/). The protein sequences were aligned using MUSCLE^71^; then the peptide alignments were back translated to DNA coding sequences using PAL2NAL^72^. Alignments were trimmed using GBLOCKS^73^. Distances were estimated using the Tamura-Nei model of evolution for a maximum-likelihood^74^ tree with the MEGA6 software package^75^. To provide statistical support for each node on the tree, a consensus tree was generated from 1000 bootstrap data sets. Protein accessions are contained in Supporting Table S2.

### Y2H analysis

Protein–protein interactions were analysed using a GAL4–based yeast hybrid system (Matchmaker two-hybrid system; Clontech). Competent cells of *Saccharomyces cerevisiae* strain AH109^76^ were transformed with pGBT9 derivatives (bait vectors; Clontech) using the LiAc/DNA/PEG transformation method^77^. The resulting yeast cells were subsequently transformed with a pGAD424 derivative (pray plasmid, Contech) dependent on the interaction pair to test. Self-activation assays and selection of positive interactors were performed according to Causier and Davies (2002)^78^.

### RNA extraction and cDNA preparation

*Arabidopsis thaliana* total RNA was extracted using Trizol reagent (TermoFisher Scientific) according to the manufacturer’s instructions. *Quercus suber* total RNA was obtained using the CTAB/LiCl extraction method^79^ with some modifications^80^ *. A. thaliana* and *Q. suber* cDNAs were synthesized according to the Invitrogen cDNA synthesis kit SuperScript® III RT manufacturer’s instructions.

### RT-qPCR analysis

cDNA was amplified using SsoFast™ EvaGreen® Supermix (Bio-Rad), 250 nM of each gene-specific primer (listed in Supplementary Table 3.1) and 1 μL of cDNA (1:100 dilution). Quantitative real-time PCR (RT-qPCR) reactions were performed in triplicates on the CFX96 Touch™ Real-Time PCR Detection System (Bio-Rad). After an initial period of 3 min at 95 °C, each of the 40 PCR cycles consisted of a denaturation step of 10 s at 95 °C and an annealing/extension step of 10 s at the gene specific primer temperature. With each PCR reaction, a melting curve was obtained to check for amplification specificity and reaction contaminations, by heating the amplification products from 60 °C to 95 °C in 5 s intervals. Primer efficiency was analysed with CFX Manager™ Software v3.1 (Bio-Rad), using the Livak calculation method for normalized expression^81^. Gene expression analysis was established based on three technical and three biological replicates, and normalized with the reference gene *QsPP2AA3* ^82^.

### Plasmids construction

Overexpression constructs were obtained using Gateway technology (ThermoFisher Scientific) according to the manufacturer’s instructions. The destination vector was pMDC32. For the yeast-two-hybrid assay, open reading frames were cloned using standard molecular biology tools in the pGBT9 or pGAD424 vectors (Clontech). Complementation constructs with native *A. thaliana* promoters were obtained by substituting the 35 S cauliflower promoter for a fragment of the *AtAP3* (0.5 Kbp) or *AtPI* (1.5 Kbp) promoters^83^ using standard molecular biology tools. All the primers used are listed in Supporting Table S1.

## Electronic supplementary material

Supplementary information file
