# Supplementary material for: Role of floral organ identity genes in the development of unisexual flowers of Quercus suber L
Source: Sci Rep. 2017 Sep 4;7:10368. doi: 10.1038/s41598-017-10732-0 (PMC5583232; doi:10.1038/s41598-017-10732-0)
Supplement: Supplementary file 1 — Supplementary information file [file 41598_2017_10732_MOESM1_ESM.docx]

**Role of floral organ identity genes in the development of unisexual flowers of *Quercus suber* L.**

Rómulo Sobral, M. Manuela R. Costa*

Biosystems and Integrative Sciences Institute (BioISI), Plant Functional Biology Center, University of Minho, Campus de Gualtar, 4710-057 Braga, Portugal.

* - corresponding author: manuela.costa@bio.uminho.pt

**Supplementary Data**

**SEM analysis and Imaging**

Samples were mounted on an aluminum stub using O.C.T. compound (Agar Scientific Ltd, Stansted, UK). The stub was then immediately plunged into liquid nitrogen slush to cryo-preserve the material. The frozen sample was transferred to the cryostage of an ALTO 2500 cryo-transfer system (Gatan UK, Abingdon) attached to an FEI Nova NanoSEM 450 FEG scanning electron microscope (FEI UK Ltd, Cambridge). Sublimation of surface frost was performed at - 95°C for 3 min before sputter-coating the sample with platinum for 2 min at 10 mA, at temperatures below -110°C. After sputter-coating, the sample was moved to the cryostage in the main chamber of the microscope, held at -125°C. The sample was imaged at 3 kV, and digital TIFF files were stored. *A. thaliana* flower photographs were taken using the Leica EZ4D HD stereo microscope.


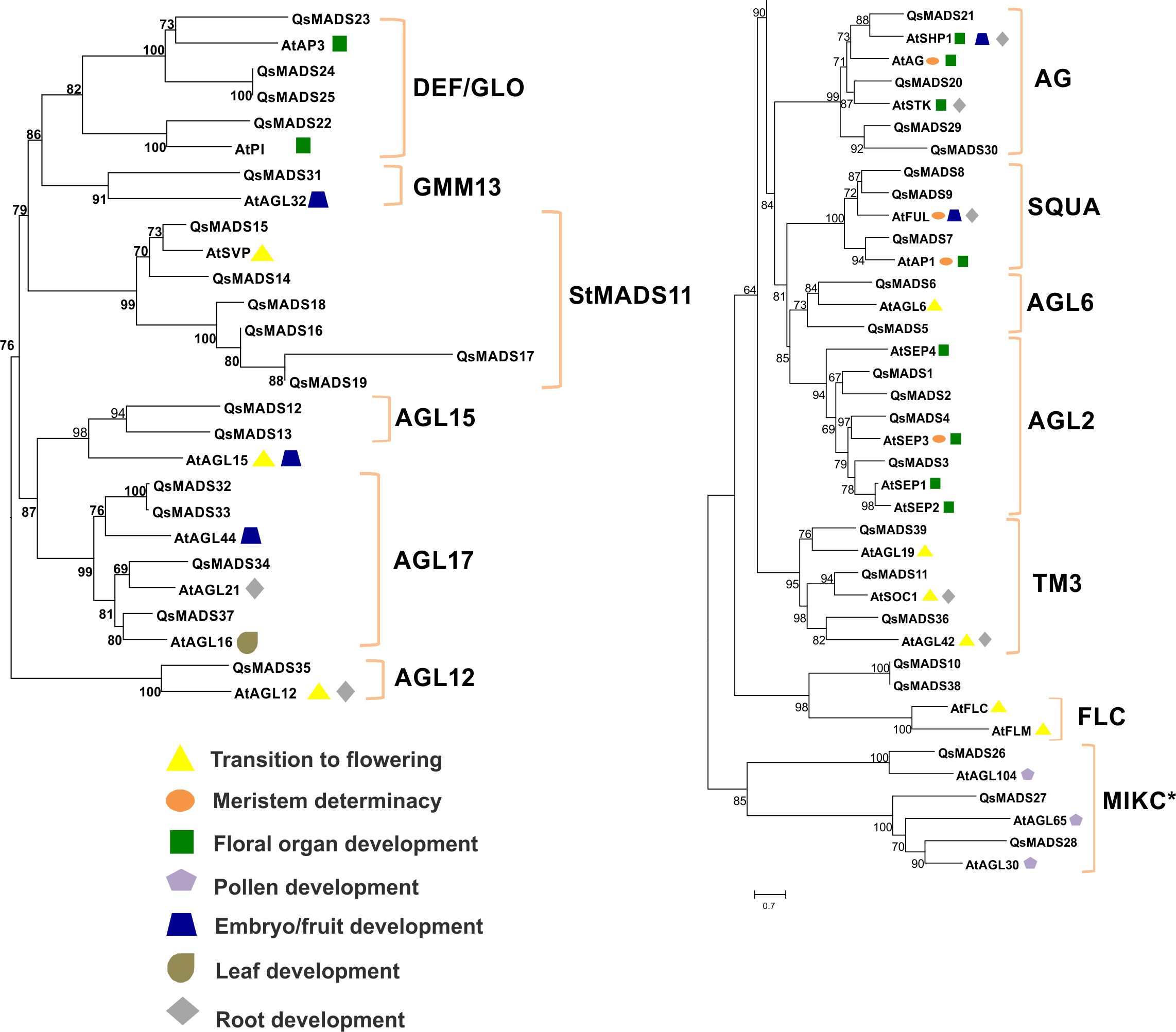


**Supplementary Figure 1 –** ***Quercus suber* MADS-box proteins and their putative function based on phylogenetic inference.** The phylogenetic relationship of the *Q. suber* MADS-box genes family was inferred using *A. thaliana* homologous proteins. MADS-box proteins clustered into fourteen phylogroups (MICK*, AGL12, GMM13, AGL17, AGL6, TM3, StMADS11, FLC, AGL15, AG, DEF/GLO, AGL2, SQUA). A symbol was added to represent a known function in plant development (yellow triangle - transition to flowering; orange circle - meristem determinacy; green square - floral organ development; purple pentagon - pollen development; blue trapezium - embryo/fruit development; brown leaf - leaf development; and grey diamond - root development).

**
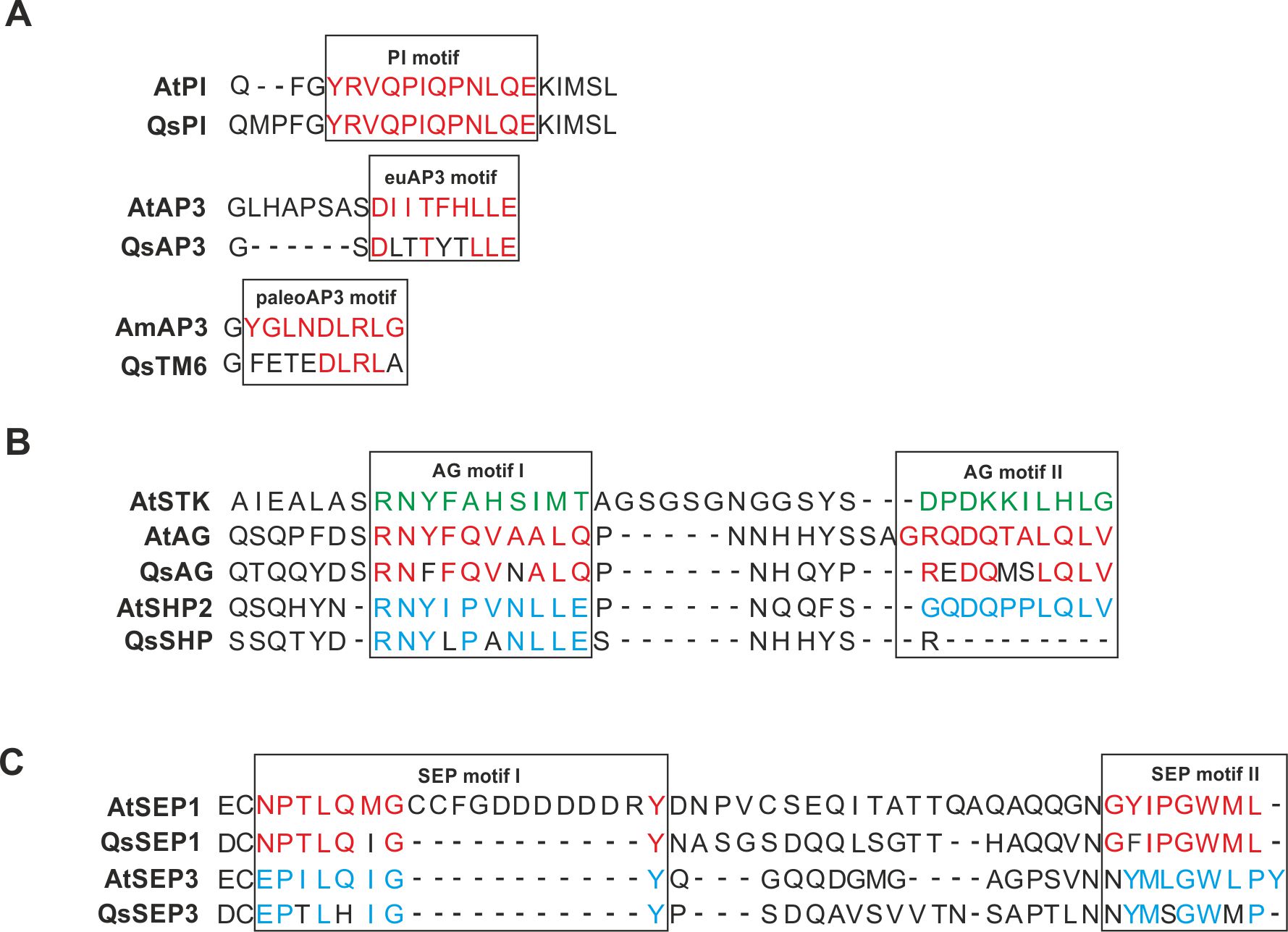
**

**Supplementary Figure 2 – BCDE lineage specific C-terminal motifs are conserved in *Q. suber* homologous proteins.** Sequence alignment of C-terminal domains of *Q. suber* and *A. thaliana* BCDE-like proteins (*Antirrhinum majus* AP3 in the case of QsTM6). **A** - B-class lineage specific motifs (euAP3, paleoAP3 and PI). **B** - C- and D-class lineages specific motifs (AG motif I and II). **C** - E-class lineages specific motifs (SEP motif I and II).

**
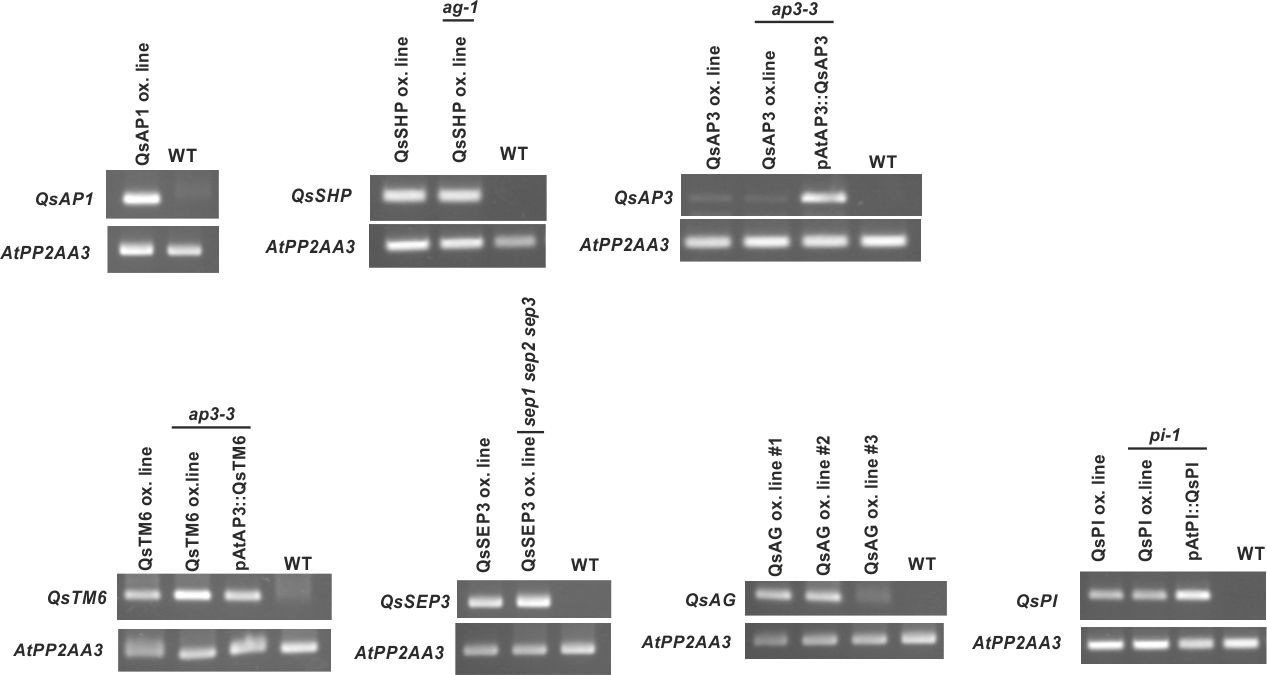
**

**Supplementary Figure 3 – *A. thaliana* plants overexpressing *QsABCDE-like* genes show different levels of the transgene expression.** Gene expression analysis by RT-PCR on *A. thaliana* plants expressing *QsAP1*, *QsTM6*, *QsAP3* *QsSHP*, *QsPI*, *QsAG* and *SEP3* under the control of the 35S promoter or the native *A. thaliana* promoter in the Col-0 or a mutant background. *AtPP2AA3* was used as reference gene. Each cropped image corresponds to a matching agarose gel.

**
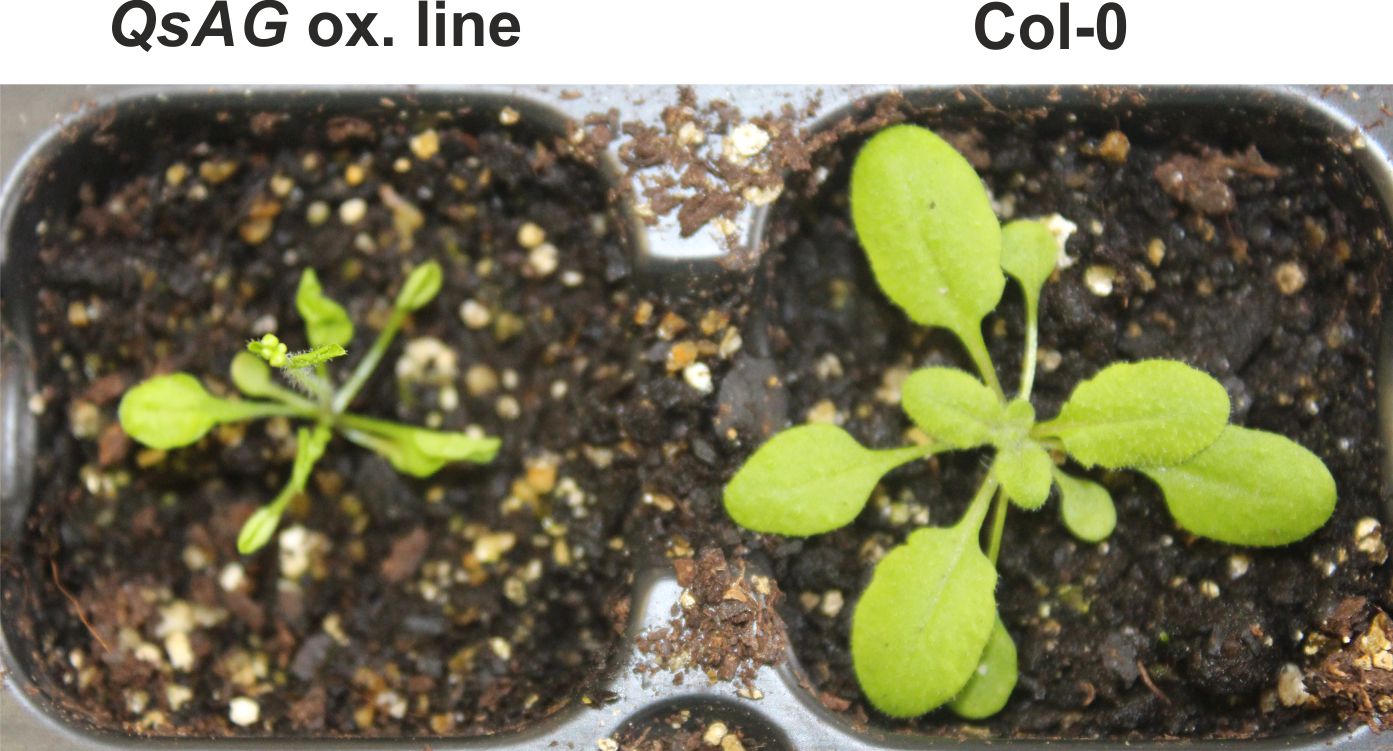
**

**Supplementary Figure 4 – *A. thaliana* plants overexpressing *QsAG* show a *curly leaf* phenotype.** Wild-type (Col-0).

**
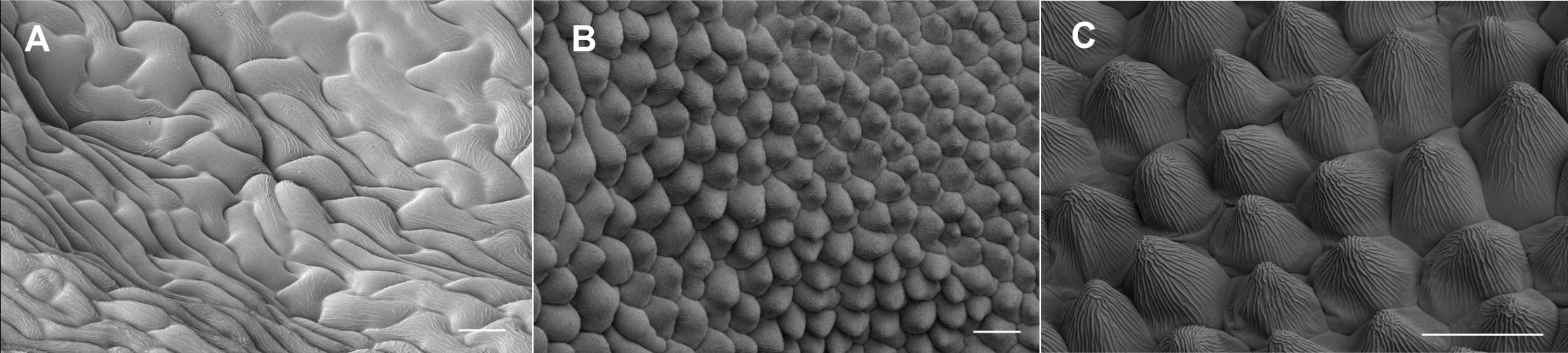
**

**Supplementary Figure 5 – Scanning electron microscopy of flower organs of *A. thaliana* overexpressing *QsPI*. A** - Wild-type sepal cells. **B** - *35S::QsPI* petal cells. **C** - *35S::QsPI* sepal cells. Scale bar: 50 μM.

**Supplementary Table 1 – List of primers**

| **Amplicon** | **Direction** | **sequence 5'-3'** | **Amplicon** | **Direction** | **sequence 5'-3'** |
| --- | --- | --- | --- | --- | --- |
| ***QsSEP1*** | Forward | CCAGCTTCCTCCTCAATCAG | ***QsSHP*** | Forward | AGGGAAGTTGAGCGCAAAA |
|  | Reverse | CTAAAGCATCCACCCAGGAA |  | Reverse | CTGGGAGGTAGTTCCGATCA |
| ***QsSEP4*** | Forward | GCAGGATTTGCCTGATTTGT | ***QsPI*** | Forward | CCGAGAAATGCAGATGGAGT |
|  | Reverse | AATGCTGCCCTTCAATCAAC |  | Reverse | AATAGGCTGCACACGGAAGG |
| ***QsSEP2*** | Forward | AGTGGAAGCAAACAGGGCTT | ***QsAP3*** | Forward | GACCGCAAGTACCAGGTGAT |
|  | Reverse | CCGCCTTCCCATGATTGTCT |  | Reverse | TGCCTAGGTCTCAGGCGTAA |
| ***QsSEP3*** | Forward | ACTGTGAACCCACGTTGCAT | ***QsTM6.1*** | Forward | CTGCTCGATCTTAGGGCAAG |
|  | Reverse | TCATGGCATCCACCCTGA |  | Reverse | AGTTGGAGGCACCATTTGTC |
| ***QsAGL6.1*** | Forward | ACCACATTGTTGGCTTGTGA | ***QsTM6.2*** | Forward | CTGCTCGATCTTAGGGCAAG |
|  | Reverse | AGCCTACGAGCTCTCTGTGC |  | Reverse | AGTTGGAGGCAGGCCATTTGTC |
| ***QsAGL6.2*** | Forward | GTGGAGAAGGATGGAAAGCA | ***QsAGL104*** | Forward | GAAGATGTGCTCGCACGTTA |
|  | Reverse | AGCAGCTTGAAGGAGCTCTG |  | Reverse | GGTTCTCAGCCATCTGAAGC |
| ***QsAP1*** | Forward | CTACCACAGCCACTTCCCTG | ***QsAGL65*** | Forward | GTTGTGTGACAGTGGGCATC |
|  | Reverse | CTATCCATTCACATGGCGAAG |  | Reverse | GCGCAGAAAAGTCTTTCACC |
| ***QsFUL.1*** | Forward | CACACCAAGAGCAACCAAGC | ***QsAGL30*** | Forward | GCCCAACCATGCTGAGTAGT |
|  | Reverse | AAGGTGGCATGACTGTAGGC |  | Reverse | CAAGAACATGGCTTGCTGAA |
| ***QsFUL.2*** | Forward | TCGCTGTAAAACCTCCATCC | ***QsSTK.1*** | Forward | ACTAGCCGGAGTGAGTTCCA |
|  | Reverse | TGCTTTGTGATGCTGAGGTC |  | Reverse | TCAGAGCCACTTCAGCTTCA |
| ***QsFLC*** | Forward | CCAGATCCAGGAAGACACAGC | ***QsSTK.2*** | Forward | CATGCTTGGACAGCTCAGAA |
|  | Reverse | CTAAGTGTTTCCAGTTGCGGC |  | Reverse | TCGAGCTCCATCACCTTCTT |
| ***QsSOC1*** | Forward | GCAGCTGAAAATGCAAGGCT | ***QsAGL32*** | Forward | AAGTCCTCACCGGTGTAACG |
|  | Reverse | GCGCTTTGTTCTCCCTTCT |  | Reverse | CCCAAATTGGAATCATGGTC |
| ***QsAGL15*** | Forward | CATTTGGTTCCCTGAGTCGT | ***QsAGL44.1*** | Forward | AAAGTCACTTGCCTGCTGGT |
|  | Reverse | GCCAATCATGGTGTTGTGAG |  | Reverse | CCGATTGGGGTATCATTTTG |
| ***QsAGL18*** | Forward | CCCAAGAATCCCACCCTATT | ***QsAGL44.2*** | Forward | TCAAGTTTGCTGCCTCCTTT |
|  | Reverse | CTTTGGCCTTCTTGAGCAAC |  | Reverse | GGAGCTCTCGATCCTTTGTG |
| ***QsSVP1*** | Forward | GGACTTACCCGTGTGCTTGA | ***QsAGL21*** | Forward | TCCCTCTGCCAAAACTTGAC |
|  | Reverse | ATGTCCGAGTCCACAAGACC |  | Reverse | TGATGCTGAAGTTGGACTCG |
| ***QsSVP3*** | Forward | TCGAATTCGCCAGCTCAAGT | ***QsAGL12*** | Forward | TATGGCTCGTGGAAAGGTTC |
|  | Reverse | TGCTCAACATGGCGTAGGAG |  | Reverse | CAATAAGCCCTTGCATGGTT |
| ***QsSVP2*** | Forward | AATCTGGATTGAGCCGTGTG | ***QsAGL42*** | Forward | TAGAGCGAAAAGAGGGCAAA |
|  | Reverse | TCAGTGCCAATGTGTCTTCG |  | Reverse | ATGAGGAAACGGGTTCTGTG |
| ***QsSVP4*** | Forward | TCTCTGCGATGCTGAAATTG | ***QsAGL16*** | Forward | TGAATTTGGTTTCCCTTTCG |
|  | Reverse | CTCAACATGGCgTAGGAGGT |  | Reverse | CAAGAAAACCATCGGCAAAT |
| ***QsSVP5*** | Forward | AAAATGGCGAGGAGGAAAAT | ***QsAGL27*** | Forward | GGTCAAAGTCAGCTCGAAGG |
|  | Reverse | GCTGCTGCATACTTGAGCTG |  | Reverse | CGTCGCTCTGATCTTCCTCT |
| ***QsSVP6*** | Forward | GCCATGTTCAGCAAGGAAAT | ***QsAGL19*** | Forward | GACCAAGGACTCAACGGAAA |
|  | Reverse | ATCAGTTGGGCTCCCTTTTT |  | Reverse | CTTCGCTCCAACTGGTTCTC |
| ***QsAG*** | Forward | CCAGCTTCTCCGAGCAAAGA | ***QsPP2AA3*** | Forward | GGGTTCCCAACATCAAGTTC |
|  | Reverse | CATCTGGTCTTCACGTGGGT |  | Reverse | TGACCTGATCACTTGACTGC |
| ***QsAP1 Y2H*** | Forward | AGGATCCAAATGGGAAGGGGTAGGGTTCA | ***QsAP3 gtw*** | Forward | AAAAAGCAGGCTTTACAATGGCAAGGGGAAAGATTCA |
|  | Reverse | AACTGCAGTCATGTGGCAAAGCATCCAAG |  | Reverse | AGAAAGCTGGGTTGCTACTCAAGCAAGGTGTAAG |
| ***QsPI Y2H*** | Forward | AGGATCCAAATGGGGAGAGGCAAGATTGAG | ***QsPI gtw*** | Forward | AAAAAGCAGGCTTTACAATGGGGAGAGGCAAGATTGA |
|  | Reverse | AACTGCAGTTACATTCTCTCTTGTAGATTTGGC |  | Reverse | AGAAAGCTGGGTTTTACATTCTCTCTTGTAGATTTGG |
| ***QsAG Y2H*** | Forward | AGGATCCAAATGGTGTACCGAACCAATCC | ***QsAG gtw*** | Forward | AAAAAGCAGGCTTTACAATGGTGTATCCGAACCAATA |
|  | Reverse | AACTGCAGTTAAACTAATTGAAGAGACATCTGGT |  | Reverse | AGAAAGCTGGGTTTTAAACTAATTGAAGAGACATCTG |
| ***QsTM6 Y2H*** | Forward | AAAGTCGACAAATGGGTCGTGGAAAGATCGAG | ***QsTM6 gtw*** | Forward | AAAAAGCAGGCTTTACAATGGGTCGTGGAAAGATCGA |
|  | Reverse | AACTGCAGTCAAGCAAGGCGCAGATCC |  | Reverse | AGAAAGCTGGGTTTCAAGCAAGGCGCAGATCC |
| ***QsAP3 Y2H*** | Forward | AGGATCCAAATGGCAAGGGGAAAGATTCAGA | ***QsAP1 gtw*** | Forward | AAAAAGCAGGCTTTACAATGGGAAGGGGTAGGGTTCA |
|  | Reverse | AACTGCAGCTACTCAAGCAAGGTGTAAGTGG |  | Reverse | AGAAAGCTGGGTTTCATGTGGCAAAGCATCCAAG |
| ***QsSEP1 Y2H*** | Forward | AGGATCCAAATGGGGAGGGGAAGAGTTGAG | ***QsSEP3 gtw*** | Forward | AAAAAGCAGGCTTTACAATGGGTAGAGGAAGAGTGGA |
|  | Reverse | AACTGCAGTCAAAGCATCCAACCAGGAATG |  | Reverse | AGAAAGCTGGGTTTCATGGCATCCACCCTGA |
| ***QsSEP3 Y2H*** | Forward | AGGATCCAAATGGGTAGAGGAAGAGTGGAG | ***QsSHP gtw*** | Forward | AAAAAGCAGGCTTAACAGAGGGATCTTCGCAGAGGAA |
|  | Reverse | AACTGCAGTCATGGCATCCACCCTGA |  | Reverse | AGAAAGCTGGGTTTTAGCGAGAGTAATGATGATTGGA |
| ***QsSHP Y2H*** | Forward | AGGATCCAACTGGAGGGATCTTCGCAGAG | ***pAtAP3 comple*** | Forward | TTAAAAGCTTAGTGACGATTAATCCAAACATATAT |
|  | Reverse | AACTGCAGTTAGCGAGAGTAATGATGATTGGA |  | Reverse | GGCGCGCCATTCTTCTCTCTTTGTTTAATCTTTTT |
| ***AtPP2AA3*** | Forward | GCGGTTGTGGAGAACATGATACG | ***pAtPI comple*** | Forward | TTAAAGCTTGAAAATTAGTCATCTTCTTCATCC |
|  | Reverse | AACCAAACACAATTCGTTGCTG |  | Reverse | GGCGCGCCCTTGTCTCTCTCTATCTCTTTCT |

**Supplementary Table 2 – List of gene accessions**

| **Cork oak** | **Corkoak database** | ***Arabidopsis thaliana*** | **NCBI accession** | ***Malus domestica*** | **NCBI accession** |
| --- | --- | --- | --- | --- | --- |
| *QsSEP1* | QSP108316.0 | *AtSEP2* | AAA32732.1 | *MdAP1* | ABG85297.1 |
| *QsSEP4* | QSP022774.0 | *AtAGL6* | ABK28537.1 | *MdAG* | CAC80858.1 |
| *QsSEP2* | QSP061360.0 | *AtSEP4* | AEC05738.1 | *MdTM6* | BAC11907.1 |
| *QsSEP3* | QSP030785.0 | *AtSEP1* | P29382.2 | *MdPI* | CAC28022.1 |
| *QsAGL6.1* | QSP158943.0 | *AtSEP3* | P22456.1 | *MdSEP3* | ADL36740.1 |
| *QsAGL6.2* | QSP040694.0 | *AtAP1* | P35631.2 | *MdSEP1* | AAC25922.1 |
| *QsAP1* | QSP003005.0 | *AtFUL* | Q38876.1 | ***Vitis vinifera*** | **NCBI accession** |
| *QsFUL.1* | QSP083983.0 | *AtFLC* | AED91498.1 | *VvAP1* | ACZ26528.1 |
| *QsFUL.2* | QSP020922.0 | *AtSOC1* | AEC10583.1 | *VvAG* | CBI30760.3 |
| *QsFLC* | QSP044604.0 | *AtAGL15* | Q38847.1 | *VvTM6* | NP_001267937.1 |
| *QsSOC1* | QSP149164.0 | *AtSVP* | AEC07320.1 | *VvAP3* | CBI19790.1 |
| *QsAGL15* | QSP048803.0 | *AtAG* | AEE84111.1 | *VvPI* | AAY79173.1 |
| *QsAGL18* | QSP143328.0 | *AtSHP1* | AEE79831.1 | *VvSEP3* | CBI27678.1 |
| *QsSVP1* | QSP116365.0 | *AtPI* | P48007.1 | *VvSEP1* | CBI16936.1 |
| *QsSVP3* | QSP055926.0 | *AtAP3* | P35632.1 | *VvSHP* | AKK58564.1 |
| *QsSVP2* | QSP020714.0 | *AtAGL104* | AAF86555.1 | ***Solanum***  ***lycopersicum*** | **NCBI accession** |
| *QsSVP4* | QSP012535.0 | *AtAGL65* | AAN37407.1 | *SlAP1* | NP_001234665.1 |
| *QsSVP5* | QSP056153.0 | *AtAGL30* | AEC05660.2 | *SlAG* | XP_004232995.1 |
| *QsSVP6* | QSP095201.0 | *AtSTK* | ANM67809.1 | *SlTM6* | CAJ53871.1 |
| *QsAG* | QSP034737.0 | *AtAGL32* | ANM68676.1 | *SlPI* | ABG73411.1 |
| *QsSHP* | QSP001472.0 | *AtAGL44* | CAB09793.1 | *SlSEP3* | AAP57412.1 |
| *QsPI* | QSP071214.0 | *AtAGL21* | AAL73213.1 | *SlSEP1* | AAM33104.1 |
| *QsAP3* | QSP122988.0 | *AtAGL12* | AAC49085.1 | ***Cucumis sativus*** | **NCBI accession** |
| *QsTM6.1* | QSP012523.0 | *AtAGL42* | AAL06880.1 | *CsAP1* | XP_011650331.1 |
| *QsTM6.2* | QSP078807.0 | *AtAGL16* | ABN04784.1 | ***Oryza sativa*** | **NCBI accession** |
| *QsAGL104* | QSP120571.0 | *AtAGL19* | AEE84684.1 | *OsAP1* | XP_015631033 |
| *QsAGL65* | QSP093997.0 | ***Populus tricocharpa*** | **NCBI accession** | *OsAG* | XP_015632490.1 |
| *QsAGL30* | QSP088783.0 | *PtAP1* | EEE88720.1 | *OsAP3* | AER42130.1 |
| *QsSTK.1* | QSP145035.0 | ***Castanea molissima*** | **NCBI accession** | *OsPI* | EEC79255.1 |
| *QsSTK.2* | QSP010209.0 | *CmAP1* | AAZ77749.1 | *OsSEP* | QOJ466.2 |
| *QsAGL32* | QSP017336.0 | *CmAG* | AAZ77747.1 | ***Petunia x hybrida*** | **NCBI accession** |
| *QsAGL44.1* | QSP130134.0 | ***Antirrhinum majus*** | **NCBI accession** | *PhTM6* | CAA49567.1 |
| *QsAGL44.2* | QSP043658.0 | *AmFAR* | CAB42988.1 | *PhPI* | CAA50549.1 |
| *QsAGL21* | QSP118715.0 | *AmPLE* | BAI68391.1 | *PhAP3* | AAQ72510.2 |
| *QsAGL12* | QSP078099.0 | ***Pinus radiata*** | **NCBI accession** | *PhAG* | CAA51417.1 |
| *QsAGL42* | QSP131838.0 | *Pinus AP1* | AAD09207.1 | *PhSHP* | CAA48635.1 |
| *QsAGL16* | QSP146997.0 | *Pinus CD* | ADD09342.1 | *PhSEP1* | AAQ72498.1 |
| *QsAGL27* | QSP081167.0 | *Pinus B* | AAF28863.1 | *PhSEP3* | AAA86854.1 |
